# A retrospective cohort study on European Reference Network for Rare Vascular Diseases 5 outcome measures for Hereditary Haemorrhagic Telangiectasia in Denmark

**DOI:** 10.1186/s13023-021-02160-1

**Published:** 2022-01-06

**Authors:** Troels Hvelplund, Bibi Lange, Susanne Djernes Bird, Malene Korsholm, Anette Drøhse Kjeldsen

**Affiliations:** 1 Research Unit for ORL – Head and Neck Surgery and Audiology, Odense University Hospital, J.B. Winsløws Vej 4, 1st Floor, 5000 Odense C, Denmark; 2 University of Southern Denmark, Odense, Denmark; 3 Open Patient Data Explorative Network (OPEN), Department of Clinical Research, University of Southern Denmark and Odense University Hospital, Odense, Denmark

**Keywords:** Hereditary Haemorrhagic Telangiectasia, VASCERN, PAVM screening, Iron deficiency

## Abstract

**Background:**

Hereditary Haemorrhagic Telangiectasia (HHT) is an autosomal dominant disorder characterized by several clinical symptoms including epistaxis, arteriovenous malformations (AVM), and telangiectasia. In 2018, European Reference Network for Rare Vascular Diseases (VASCERN) recommended five outcome measures for HHT-patients to guide health care providers, some with limited experience in treating HHT, and thereby maximizing the number of HHT-patients receiving good care. The outcome measures cover the following aspects: (1) 90% of the patients should receive a pulmonary AVM (PAVM) screening; (2) 90% of the patients should receive written advice on nosebleed; (3) 70% should be assessed for iron deficiency; (4) 100% of the patients should receive written advice on antibiotic (AB) prophylaxis prior to dental and surgical procedures, and (5) 100% of relevant patients should receive written advice on pregnancy. We have introduced the outcome measures as Benchmarks in our HHT-centre and wanted to evaluate the extend of implementation we have achieved. We constantly struggle to secure the best possible treatment of our HHT-patients.

**Methods:**

The study was a non-interventional retrospective study. Data was collected manually from patient records and from the Danish HHT-database.

**Results:**

A total of 180 HHT-patients were included, all diagnosed in the period from January 1st, 2016, to December 31st, 2020. All patients were screened for PAVM. We could confirm that 66% of patients who had epistaxis received thoroughly advice. Assessment for iron deficiency was performed in 80% of the adult patients. Thoroughly advice on AB prophylaxis was documented in 75%. Thoroughly advice on pregnancy was documented in 80% of female patients 15–45 years of age. There were no significant differences over time for any of the outcome measures.

**Conclusions:**

The Danish HHT-centre reached the target threshold for outcome measures 1 and 3. It could not be documented that the target thresholds for outcome measures 2, 4, and 5 were achieved. As information and education are a very important part of HHT care, focus on and documentation that all patients receive the relevant advice must be a priority in order to ensure best care.

## Introduction

HHT is a rare and autosomal dominant inherited disease that affects approximately 1 out of 6,400 [1]. HHT affects growth and repair of endothelial cells in the capillaries and is characterized by dilation of the small capillaries (telangiectasia) in the skin and mucosa, as well as formation of arteriovenous malformations (AVM). AVM can occur in several different organs including pulmonary (PAVM), hepatic, and cerebral AVMs. As a result of the change in capillary anatomy, bleeding from telangiectatic lesions in the skin and the mucous membranes often occur. Clinical manifestations of HHT include recurrent epistaxis, gastrointestinal bleeding, iron deficiency anaemia, brain abscess and focal neurological symptoms [2]. Pregnancy in female patients with HHT is considered a high risk pregnancy, due to rare major complications like PAVM bleed and maternal death [3].

European Reference Network on Rare Vascular Diseases (VASCERN) is an EU supported collaboration among European hospitals. One of VASCERNs goals is to encourage the development of quality and safety benchmarks, and help to develop and implement best practice [4]. In 2018, VASCERN published a position statement regarding 5 outcome measures in order to maximize the number of HHT-patients receiving good care [5]. The outcome measures were evaluated as “easy to use”, also for healthcare providers with limited training in how to handle HHT-patients.

The 5 outcomes cover the following areas: (1) pulmonary screening; (2) written nosebleed advice; (3) assessment of iron deficiency anaemia; (4) written advice on AB prophylaxis prior to dental or surgical procedures and (5) written advice on pregnancy.

The Danish HHT-centre is located at Odense University Hospital and is a part of VASCERN. The centre is responsible for diagnostics and treatment of all HHT-patients in Denmark. Furthermore, the centre manages a database of all HHT-patients.

To our knowledge, no study has investigated the implementation of VASCERN recommendations at any hospital in Europe (Appendix 1, search strategy). The aim of this study is to investigate the degree of implementation of the VASCERN 5 outcome measures for HHT-patients.

## Methods

The study was a non-interventional retrospective cohort study of all patients diagnosed with HHT, at the Danish HHT-centre, in the time period January 1st, 2016, to December 31st, 2020. The patients were identified using the HHT-database. Study data of HHT-patients concerning PAVM screening, mutation diagnostics, age and sex was collected from the HHT-database. Data regarding information given to the patients as well as data on anaemia was extracted from patient records. Data was managed using Research Electronic Data Capture (REDCap) [6] with access provided by OPEN [7]. HHT was diagnosed either by a molecular genetic test or clinically using the Curaçao criteria [8].Group I consisted of the patients visiting the Danish HHT-centre before the VASCERN recommendations, and group II consisted of the patients visiting the centre after the recommendations were published (February 12th 2018). As part of the assessment of information, a small qualitative study (telephone interview) was performed in a group of newly diagnosed HHT-patients.

### Aim

The objective of the study was to evaluate if:

- At least 90% of patients had a screening for PAVM.
  - Secondary, to investigate the prevalence of PAVM among different HHT-genotypes, and if PAVMs were treated.
- At least 90% of patients received written advice on nosebleed.
- At least 70% of patients were assessed for iron deficiency anaemia.
  - Secondary, to investigate the prevalence of anaemia in newly diagnosed HHT-patients
- 100% of all patients with PAVM received written advice on prophylactic AB.
- 100% of patients received written advice on pregnancy.

Patient records were scanned and categorized by TH. In case of doubt, ADK was consulted to determinate the right category. For further detail information about categorization, see Appendix 2.

### Screening for PAVM

At the HHT-centre OUH three screening modalities are used: first measurement of oxygen saturation (SAT), secondly a Transthoracic Contrast Echocardiography (TTCE) and if the TTCE is pathological a Computer Tomography (CT) -scanning of the chest is offered. In order to identify a pathological TTCE a grading system is used to categorize the severity of a shunt. Shunts are graded from 0 to 4, with 4 being the most severe. Studies have shown that an increased shunt grade predicts an increased probability for PAVM [9, 10]. At the Danish HHT-centre, only patients with a TTCE grade of 2, 3 or 4 are offered a thoracic CT-scan. We are not aware of PAVM in patients with TTCE grade 0 or 1, and to reduce the amount of x-ray in patients, CT-scanning for TTCE grad 0 and 1 are not performed.

TTCE scanning is offered to all patients over the age of 12 years. If a patient has a TTCE between the age 12 and 17, a new TTCE is offered at the age of 18 years when the child is fully grown. Likewise, all women are offered a TTCE after each pregnancy. If a treatable PAVM is identified the patient is referred to embolization and/or in few cases surgery.

Data regarding PAVM screening was divided into three categories: screening for PAVM was initiated; screening for PAVM was not initiated, and PAVM was already diagnosed.

PAVM status were divided into three categories: no PAVM; PAVM, or micro-PAVM.

### Need of treatment for PAVM according to HHT-genotype

To determine HHT-genotype and treatment, existing data from the HHT-database were used. In the database the genotype of HHT-patients was categorized in four groups: HHT1, HHT2, juvenile polyposis HHT (JP-HHT) and clinical HHT with no mutation (either laboratory mutation test was not able to identify a mutation or mutation diagnostics had not been performed). Treatment of PAVM was categorized in four ways: embolization, surgery, surgery and embolization and too small to treat.

### Written advice on epistaxis

Patients who had not experienced any epistaxis were categorized by “written advice not relevant” and were excluded from the analysis. This group included patients who were referred having an HHT-mutation, discovered as part of a family investigation but who had never experienced epistaxis and did not want written advice. Data on written epistaxis advice was divided into three categories: no written advice given; written advice given; the patient was informed about epistaxis. In the analysis, the two categories “written advice given” and “the patient was informed about epistaxis” were merged into one group.

### Assessment on iron deficiency anaemia

Data on assessment regarding iron deficiency anaemia was divided into two categories; whether iron deficiency anaemia was assessed at the first visit or not.

Data on iron deficiency anaemia was collected in all patients 12 years or older. This age limit was set due to the clinical practise of not routinely taking blood samples in younger children.

Low haemoglobin (Hgb) was defined as followed:

- Adults (> 18 years): Hgb < 8.3 mmol/L for men, and Hgb < 7.3 mmol/L for women
- Children (≥ 12–18 years): Hgb < 7.8 mmol/L for boys, and Hgb < 7.0 mmol/L for girls

Low ferritin was defined as followed:

- Adults (≥ 15 years): Ferritin < 15 µg/L
- Children (< 15 years): Ferritin < 12 µg/L

### Written advice on AB prophylaxis

Patients categorized by “written advice not relevant” were excluded from this analysis. This included patients who did not have any signs of PAVM at screening. Data on written advice on AB prophylaxis was divided into three categories: no written advice given; written advice given; the patient was informed about AB. In the analysis, the two categories “written advice given” and “the patient was informed about AB prophylaxis” were merged into one group.

### Written advice on pregnancy

VASCERN recommend that all pregnant women with a CT-verified PAVM should receive written advice on pregnancy and HHT. To secure that all potential pregnant women received written advice, the study included all women from the age of 15–45 years old. All other patients were categorised as “written advice not relevant” and were excluded from the analysis.

Data on written advice on pregnancy was divided into three categories: no written advice given; written advice given; the patient was informed about HHT and pregnancy. In the analysis, the two categories “written advice given” and “the patient was informed about HHT and pregnancy” were merged into one group.

### Patient interview

To secure that information provided to the patients was given and understood, The Danish HHT-centre made a small qualitative study. A semi-constructed interview was made to determinate if patients, who had received a pamphlet concerning HHT, were satisfied with the information orally as well as written. In the period from March 24th, 2017, to March 12th, 2018, 11 patients were interviewed by telephone  to evaluate their first visit at The Danish HHT-centre.

### Statistical analysis

Reports from the REDCap database were generated and manually extracted into a spreadsheet in Microsoft Excel version 15.18. All data was extracted into two different spreadsheets, independently. The spreadsheets were compared to avoid typing error. All information was anonymised in the spreadsheets.

The percentage of the fulfilment of each outcome measure was calculated for both group I and II (before and after publishing the outcome measures). The outcome measures 1–4 for group I and II were then compared using a chi-square-test to determinate a significant difference. Outcome measure 5 for group I and II were compared using Fischer’s exact test.

## Results

A full overview of the data can be found in Appendix 3.

### Patient characteristics

A total of 180 patients were included from the HHT-database. The mean age at diagnosis was 40.2 years spanning from 2 to 79 years. The patients consisted of 81 men and 99 women. There was no statistically significant difference between group I and group II in any of the five outcome measures. The results are presented as the combined numbers for the two groups. An overview of the results of all outcome measures can be seen in Table 1.

**Table 1:** Overview of outcome measures

Outcome measure	Target population	Cases (N)	Target threshold (%)	Fulfilment	Target threshold achieved
1. Screening for PAVM	All HHT-patients	180	≥ 90	100% (n = 180)	Yes
2. Written advice on epistaxis	All HHT-patients with epistaxis	151	≥ 90	66% (n = 99)^1^ 26% (n = 40)^2^ 40% (n = 59)^3^	No
3. Iron deficiency anaemia assessment	All HHT-patients and ≥ 12 years old	167	≥ 70	80% (n = 133)	Yes
4. Written advice on AB	All HHT patients, except 9 patients where PAVM was ruled out	171	100	75% (n = 129)^1^ 32% (n = 55)^2^ 43% (n = 74)^3^	No
5. Written advice about pregnancy	All HHT-patients, and female ≥ 15 and ≤ 45 years old	45	100	80% (n = 36)^1^ 24% (n = 11)^2^ 56% (n = 25)^3^	No

^1^Patients received written advice, or the patients were thoroughly informed

^2^Patients received written advice

^3^Patients were thoroughly informed

### Outcome measure 1: PAVM screening

**Fig. 1:**
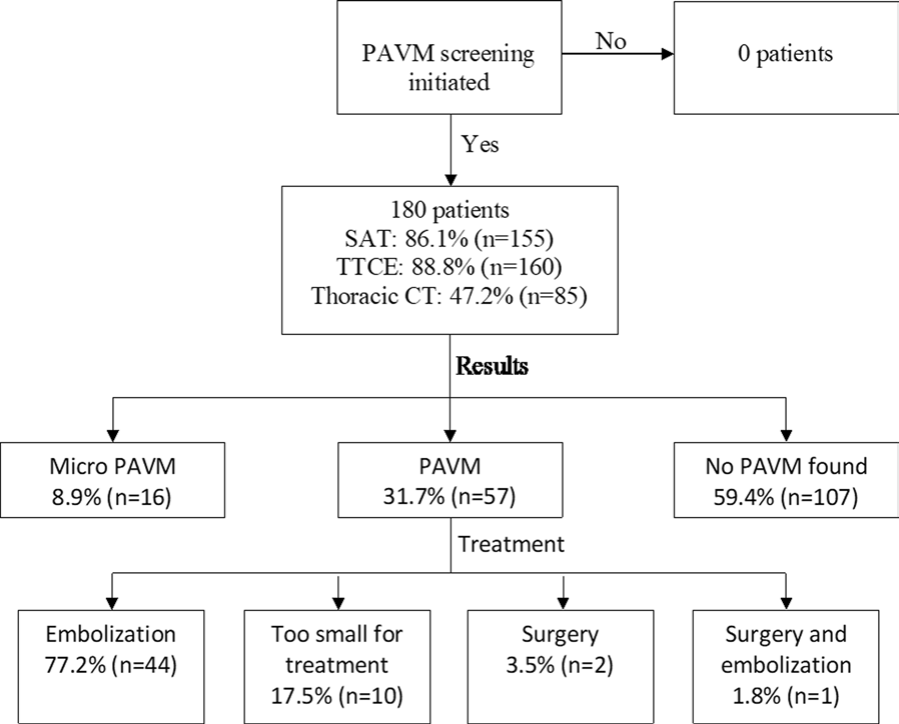
An overview of PAVM screening, the screening results, and the treatment of PAVM. *SAT* saturation measurement, *TTCE* Transthoracic Contrast Echocardiography. Patients with PAVM who were too small for treatment are followed to see if the PAVM increase in size. Surgery was in all cases performed before referral to the HHT-Centre

A total of 180 patients were included in the analysis. We found that 13% (n = 24) of the patients were already diagnosed with PAVM by a thoracic CT-scan at their first visit, and among the remaining 87% (n = 156) PAVM screening were initiated. A total of 47 (26%) patients received treatment for their PAVM thereby reducing risk for severe complications and 10 patients with PAVM too small for treatment were included in a surveillance programme (Fig. 1).

The distribution of patients with one or more PAVM between genotypes is in Table 2.

**Table 2:** Genotypes and PAVM

Genotype	Number of patients (percentage of the total population n = 180)	Patients with PAVM identified (percentage of genotype subgroup)
HHT1	89 (49%)	38 (42.7%)
HHT2	67 (37%)	12 (17.9%)
JP-HHT	15 (8%)	5 (33%)
Clinical HHT	9 (5%)	2 (22.5%)

### Outcome measure 3: assessment of iron deficiency and anaemia

A total of 167 patients were included in the analysis. Eighty percent (n = 133) were assessed for iron deficiency and anaemia. In total iron and/or Hgb deficiency was identified and treated in 34 patients. Thirteen percent (n = 17) had low levels of ferritin, and 13% (n = 17) had low levels of Hgb. In patients with either low ferritin or low Hgb, nine patients had iron deficiency and anaemia simultaneously, which corresponded to 7%.

### Patient interview

All the 11 patients interviewed by telephone had received at least one pamphlet about HHT, they all stated that they were satisfied with the information they had received.

Furthermore, one patient expressed that it was difficult to get an overview of all the tests and appointments related to the disease.

## Discussion

Although there was a tendency towards more focus on documenting the measures after publication of the outcome measures, this was not statistically significant. All the results were discussed as one unique 5-year group.

### Outcome measure 1: PAVM screening

All HHT-patients received a PAVM screening using SAT, a TTCE, or a thoracic CT-scan. The most common screening was TTCE performed in 88.8% of all patients. The scanning is not 100% since children do not receive a TTCE until they are at least 12 years of age, and a few patients declined further screening.

Due to the risk of brain abscess and ischaemic stroke associated to PAVM, detection and treatment of PAVM is important (6). Embolization has been proven to be an effective treatment for PAVM and is our prefered treatment of choice [11]. We did have 3 patients who had surgery for their PAVM, two of them before they visited our center with 1 needing further embolization therapy. One patient had PAVM and very severe pulmonary hypertension and needed lungtransplantation. In this study, we found that all patients with treatable PAVM received treatment.

The prevalence of PAVM among HHT1-patients was 42.7%. This is consistent with Letterboer et al. [12] who found that 48.7% of all HHT1-patients had PAVM. Among HHT2-patients, the prevalence of PAVM was 17.9%, while Letterboer et al. found a prevalence of 5.3%, and Abdalla et al. [13] found a prevalence of 5%. The reason for this difference is not clarified. In this study, the screening identified 47 patients with PAVM, who were treated to prevent potential life-threatening complications.

### Outcome measure 3: assessment of iron deficiency anaemia

The target threshold of 70% was achieved as 80% of the relevant population was assessed. This study investigated whether iron deficiency anaemia was assessed at the first visit, though VASCERN recommend evaluation at every visit. If patients at the Danish HHT-centre have normal ferritin and Hgb levels at their first visit, they are advised to have assessment of iron deficiency anaemia at their general practitioner (GP) e.g., once a year or if bleeding worsen. We were not able to include results from assessment at the GP in the present study.

Before their first visit at our centre, some patients may have had an iron deficiency anaemia assessment at their GP or at a hospital in a region other than the Region of Southern Denmark. This may have caused an underestimation of the number of patients who received an iron deficiency anaemia assessment. Iron and/or Hgb deficiency was identified and treated in 43 patients, helping these patients reducing fatigue and achieving a higher level of energy.

### Outcome measures 2, 4 and 5: written advice on epistaxis, AB, and pregnancy

The target threshold for outcome measures 2, 4 and 5 was not achieved in either of the measures. The outcome measures involved written advice. It was difficult to determinate if pamphlets were handed out as there was no procedure for registration. In many cases, the patient record stated that the patient was thoroughly informed about HHT in general or about epistaxis, AB prophylaxis or pregnancy specifically but without a firm registration of handing out written advice. Pamphlets are accessible at the HHT-centre and online at the HHT-centre homepage (https://www.ouh.dk/wm487733). However, there is no routine for documenting distribution of pamphlets to the patients. This may lead to an underestimation of the number of patients who have received written advice. If routines were made to document if written advice was given, it would reduce the underestimation and provide a more exact answer on number of patients receiving written advice.

In this study, we decided to investigate if female patients between 15 and 45 years of age had received written advice on pregnancy. We chose this age group as most pregnancies occur inside these margins. However, in clinical practice, pregnancy is discussed in all relevant cases and written advice is given on request also to women outside of this age group.

### Patient interviews

The patient interviews support that the real number of patients receiving written advice, are higher than found in this study. Furthermore, the patient interviews showed that the patients were satisfied with the information they received. Patients with chronic diseases and low health literacy are more likely to miss appointments and less likely to take care of their diseases and follow their treatment. Therefore, it is important that patients receive and understand the information given to them. Outcome measure 2, 4, and 5 all involve written advice about the handling of symptoms of the illness and prevention of disease deterioration. Education of patients is very important to help them understand their illnesses and enable them to make appropriate decisions about their health. Handing out pamphlets is a way to educate patients. However, this may not be sufficient. To help patients to navigate in the Danish health care system, a smartphone application (app) “My Hospital” has been developed. The app provides the patients with information about their illness, and in the future the app will further help patients manage their illness and entails written information of HHT.

Furthermore, patients must be informed that written material is available on the HHT-centres own website as well as on the patient association for HHT-patients’ website.

### Strengths and limitations

The strength of this study is inclusion of all patients with HHT registered at the Danish HHT-centre, and the study is representative for HHT-patients in Denmark.

A limitation of this study was that data retrieval from patient records was performed by only one person. Furthermore, this study relies on accurate recordkeeping concerning handing out written advice. We recommend using the 5 outcome measures, as a help for the clinicians in securing appropriate evaluation for two of the most severe HHT complications and they help to educate patients. We also recommend that patients with HHT are referred to the HHT-centre for further highly specialised evaluation regarding other aspects of HHT including mutation diagnostics and information regarding eventual screening for arteriovenous malformations in the brain and the liver.

## Conclusion

The Danish HHT-centre reached the target threshold for outcome measure 1; that 90% of the patients should receive a PAVM screening. All patients received a PAVM-screening. The screening programme is effective, resulting in high number of patients receiving proper treatment for PAVM. Target threshold for outcome measure 3; that 70% should be assessed for iron deficiency, was also achieved and help identify anaemia and iron deficiency. Outcome measures 2, 4, and 5, regarding that 90% of the patients should receive written advice on nosebleed; 100% of the patients should receive written advice on AB prophylaxis, and 100% of relevant patients should receive written advice on pregnancy, were not reached by the centre. We aim to fulfil all the outcome measures as we believe they represent valuable information and education of the patients and will increase their quality of life. Therefore, we have developed an app. which will help the HHT-patient receive written information at the most convenient time and place. Further, we will focus on education of nurses and doctors and their documentation of information to HHT-patients.

## Data Availability

Data from REDCap are not publicly available due to personal data on patients.

## Appendix 1: Search strategy

The following search string was used on PubMed – 18 hits:

((VASCERN OR European Reference Network for Rare Vascular Diseases) AND (HHT OR hereditary haemorrhagic telangiectasia OR Mb. Osler-Weber-Rendu syndrome OR morbus Osler))

Articles were screened on titles. Relevant articles were reviewed on abstract.

## Appendix 2: Detailed information about categorization

**Table:**

Outcome measure	Categories	Checked if,
Screening for PAVM	PAVM screening was initiated	Saturation had been measured, or; Patient was referred to a TTCE or; Patient was referred to a thoracic CT-scan, or; Previous thoracic CT-scan was reassessed
	PAVM screening was not initiated	No action was taken to screen or planning a screening for PAVM
	PAVM was already diagnosed at the first visit	Patients with PAVM, verified by a thoracic CT-scan upon their first visit
PAVM status	No PAVM	TTCE showed grade 0 or 1, or; Thoracic CT-scan showed no PAVM
	Micro-PAVM	TTCE showed grade 2 or 3, but thoracic CT-scan showed no PAVM
	PAVM	Thoracic CT-scan showed one or more PAVM
	PAVM not fully examined	Not fully grown children TTCE booked, but not yet completed TTCE grade 2, 3, or 4 but thoracic CT-scan not yet completed
Written advice on epistaxis	No written advice given	Patient had epistaxis, but no pamphlet was handed out and it does not appear from the patient record if the patient was informed about epistaxis
	Written advice given	Pamphlet regarding epistaxis was handed out
	The patient was informed about epistaxis	It appeared from the patient record that the patient was informed about the HHT in general or epistaxis specifically, but no pamphlet was handed out
	Written advice was not relevant	Patients who had not experienced any epistaxis
Iron deficiency anaemia	Assessed at the first visit	Ferritin and Hgb were measured related to the visit at the HHT-centre, or: Hgb and ferritin was assessed from other blood samples, not older than 3 months No exact values for ferritin or Hgb was found, but the patient record stated that an assessment had been made
	Not assessed at the first visit	Only ferritin was assessed, or; Only Hgb was assessed, or; Neither Hgb nor ferritin were assessed
Written advice on AB	No written advice given	Patients had PAVM or PAVM was not excluded, but no pamphlet was handed out, and patient record does not state if the patient was informed about AB
	Written advice given	Pamphlet regarding AB (Mb. Osler/HHT) was handed out, or; A letter from the doctor to the patient with AB advice was sent
	The patient was informed about AB	It appeared from the patient record that the patient was informed about the HHT in general or AB specifically, but no pamphlet was handed out
	Written advice was not relevant	Patients had been verified without PAVM upon their first visit
Written advice about pregnancy	No written advice given	Patient was female between 15 and 45 years old, and pamphlet regarding pregnancy and HHT was not handed out, nor was the patient informed about HHT in general or pregnancy specifically
	Written advice given	Patient was female between 15 and 45 years old, and pamphlet regarding pregnancy and HHT was handed out
	The patient was informed about pregnancy and HHT	It appeared from the patient record that the patient was informed about HHT in general or pregnancy specifically, but no pamphlet was handed out
	Written advice was not relevant	Patient was male, or; Female ≤ 15 or ≥ 45 years old

## Appendix 3: Data overview

**Table:**

	Group I (n = 70)	Group II (n = 110)	All patients (n = 180)
**Outcome measure 1: PAVM screening, results and genotypes**			
PAVM screening was initiated at the first visit	87.1% (n = 61)	86.4% (n = 95)	86.6% (n = 156)
PAVM screening was not initiated at the first visit	0.00% (n = 0)	0.00% (n = 0)	0.00% (n = 0)
PAVM was already diagnosed at the first visit	12.9% (n = 9)	13.6% (n = 15)	13.3% (n = 24)
SAT	85.7% (n = 60)	86.4% (n = 95)	86.1% (n = 155)
TTCE	87.1% (n = 61)	90% (n = 99)	88.8% (n = 160)
Thoracic CT	45.7% (n = 32)	48.2% (n = 53)	47.2% (n = 85)
Micro PAVM	4.3% (n = 3)	11.8% (n = 13)	8.9% (n = 16)
PAVM	32.9% (n = 23)	30.9% (n = 34)	31.7% (n = 57)
No PAVM found	62.9% (n = 44)	57.2% (n = 63)	59.4% (n = 107)

	Group I (n = 23)	Group II (n = 34)	All patients (n = 57)
**CT-verified PAVM and treatment**			
Embolization	65.2% (n = 15)	85.3% (n = 29)	77.2% (n = 44)
Too small for treatment	26.1% (n = 6)	11.8% (n = 4)	17.5% (n = 10)
Surgery	4.3% (n = 1)	2.9% (n = 1)	3.5% (n = 2)
Surgery and embolization	4.3% (n = 1)	0.0% (n = 0)	1.8% (n = 1)

	Group I (n = 70)	Group II (n = 110)	All patients (n = 180)
**Outcome measure 2: written advice on epistaxis**			
Written advice given	27.1% (n = 19)	19.1% (n = 21)	22.2% (n = 40)
The patient was informed about epistaxis	30.0% (n = 21)	34.5% (n = 38)	32.7% (n = 59)
No written advice given	34.3% (n = 24)	25.5% (n = 28)	28.8% (n = 52)
Written advice was not relevant	8.6% (n = 6)	20.9% (n = 23)	16.1% (n = 29)

	Group I (n = 64)	Group II (n = 87)	All patients (n = 151)
**Outcome measure 2: written advice on epistaxis.**^**1**^** Without patients categorized in “Written advice was not relevant”**			
Written advice given or the patient was informed about epistaxis	62.5% (n = 40)	67.8% (n = 59)	65.6% (n = 99)
No written advice given	37.5% (n = 24)	32.2% (n = 28)	34.4% (n = 52)

	Group I (n = 64)	Group II (n = 103)	All patients (n = 167)
**Outcome measure 3: assessment on iron deficiency anaemia**^**2**^**. All patients ≥ 12 years**			
Assessed at the first visit	82.8% (n = 53)	77.6% (n = 80)	79.6% (n = 133)
Not assessed at the first visit	17.2% (n = 11)	22.3% (n = 23)	20.3% (n = 34)

	Group I (n = 53)	Group II (n = 80)	All patients (n = 133)
**Outcome measure 3: results of assessment. All patients ≥ 12 years**			
Low ferritin	15.1% (n = 8)	11.3% (n = 9)	13% (n = 17)
Low Hgb	17% (n = 9)	10% (n = 8)	13% (n = 17)
Iron deficiency and anaemia	9.4% (n = 5)	5% (n = 4)	7% (n = 9)

	Group I (n = 70)	Group II (n = 110)	All patients (n = 180)
**Outcome measure 4: written advice on AB prophylaxis**			
Written advice given	35.7% (n = 25)	27.2% (n = 30)	30.6% (n = 55)
The patient was informed about AB prophylaxis	34.3% (n = 24)	45.5% (n = 50)	41.1% (n = 74)
No written advice given	25.7% (n = 18)	21.8% (n = 24)	23.3% (n = 42)
Written advice was not relevant	4.3% (n = 3)	5.5% (n = 6)	5% (n = 9)

	Group I (n = 67)	Group II (n = 104)	All patients (n = 171)
**Outcome measure 4: written advice on AB prophylaxis**^**3**^**. Without patients categorized in “Written advice was not relevant”**			
Written advice given or the patient was informed about AB prophylaxis	73.1% (n = 49)	76.9% (n = 80)	75.4% (n = 129)
No written advice given	26.9% (n = 18)	23.1% (n = 24)	24.6% (n = 42)

	Group I (n = 70)	Group II (n = 110)	All patients (n = 180)
**Outcome measure 5: written advice on pregnancy**			
Written advice given	2.9% (n = 2)	8.2% (n = 9)	6.1% (n = 11)
The patient was informed about pregnancy	14.3% (n = 10)	13.6% (n = 15)	13.8% (n = 25)
No written advice given	8.6% (n = 6)	2.7% (n = 3)	5% (n = 9)
Written advice was not relevant	74.3% (n = 52)	75.5% (n = 83)	75% (n = 135)

	Group I (n = 18)	Group II (n = 27)	All patients (n = 45)
**Outcome measure 5: written advice on pregnancy**^**4**^**. Without patients categorized in “Written advice was not relevant”**			
Written advice given or the patient was informed about pregnancy	66.7% (n = 12)	88.9% (n = 24)	80% (n = 36)
No written advice given	33.3% (n = 6)	11.1% (n = 3)	20% (n = 9)

^1^Chi-square-test: *p* = 0.49

^2^Chi-square-test: *p* = 0.42

^3^Chi-square-test: *p* = 0.57

^4^Fischer’s exact test: *p* = 0.13

## Abbreviations

AB: Antibiotics
App: Application
AVM: Arteriovenous malformations
CT: Computer tomography
GP: General practitioner
Hgb: Haemoglobin
HHT: Hereditary Haemorrhagic Telangiectasia
JP: Juvenile polyposis
OPEN: Open patient data explorative network
PAVM: Pulmonary arteriovenous malformations
REDCap: Research Electronic Data Capture
SAT: Saturation measurement
TTCE: Transthoracic Contrast Echocardiography
VASCERN: European Reference Network for Rare Vascular Diseases
